# Effects of a Pain Catastrophizing Induction on Sensory Testing in Women with Chronic Low Back Pain: A Pilot Study

**DOI:** 10.1155/2017/7892494

**Published:** 2017-02-28

**Authors:** Chloe J. Taub, John A. Sturgeon, Kevin A. Johnson, Sean C. Mackey, Beth D. Darnall

**Affiliations:** Division of Pain Medicine, Department of Anesthesiology, Perioperative and Pain Medicine, Stanford Systems Neuroscience and Pain Laboratory, Stanford University School of Medicine, 1070 Arastradero, Suite 200, MC 5596, Palo Alto, CA 94304-1336, USA

## Abstract

Pain catastrophizing, a pattern of negative cognitive-emotional responses to actual or anticipated pain, maintains chronic pain and undermines response to treatments. Currently, precisely how pain catastrophizing influences pain processing is not well understood. In experimental settings, pain catastrophizing has been associated with amplified pain processing. This study sought to clarify pain processing mechanisms via experimental induction of pain catastrophizing. Forty women with chronic low back pain were assigned in blocks to an experimental condition, either a psychologist-led 10-minute pain catastrophizing induction or a control (10-minute rest period). All participants underwent a baseline round of several quantitative sensory testing (QST) tasks, followed by the pain catastrophizing induction or the rest period, and then a second round of the same QST tasks. The catastrophizing induction appeared to increase state pain catastrophizing levels. Changes in QST pain were detected for two of the QST tasks administered, weighted pin pain and mechanical allodynia. Although there is a need to replicate our preliminary results with a larger sample, study findings suggest a potential relationship between induced pain catastrophizing and central sensitization of pain. Clarification of the mechanisms through which catastrophizing affects pain modulatory systems may yield useful clinical insights into the treatment of chronic pain.

## 1. Introduction

More than 100 million Americans [1] and approximately 1.5 billion people in the world [2] suffer from chronic pain, with chronic low back pain being the leading cause of disability globally [3]. Chronic pain has a profound negative impact across many health and wellness domains including physical function, mental health, quality of life, and work productivity [4]. Chronic back pain is challenging for clinicians and researchers, due in part to the potentially multifactorial etiology of pain. Many patients report significant pain and disability, but clear evidence of physical abnormalities that explain the onset and maintenance of pain is often absent [5]. Consequently, modern clinical models of pain have evolved to include other potential causes and contributors to pain, such as psychological factors and aberrancies in central nervous system function. The intimate interaction between the physical and the psychological components of the pain experience has been well documented in pain literature [6] and there remains an urgent need to better characterize the psychological factors contributing to the onset and maintenance of chronic low back pain in order to develop better-targeted and more effective treatments.

Pain catastrophizing is one psychological construct that has been shown to maintain chronic back pain [7] and to impair response to medical intervention [8]. Pain catastrophizing is a pattern of negative cognitive-emotional responses to actual or anticipated pain that includes rumination about pain, magnification of pain, and feelings of hopelessness about pain [9]. In prospective studies, pain catastrophizing has been shown to be a primary predictor of the development of chronic back pain at one year following a pain-free baseline [10] as well as a significant predictor of chronification of acute back pain [11]. Pain catastrophizing is an attractive target for intervention, as it has been successfully reduced through both multidisciplinary intervention [12] and specific catastrophizing intervention [13], and corresponds with reductions in pain intensity and other pain-related outcomes [14–16].

Currently, precisely how catastrophizing influences pain processing is not well understood, though extant theories have highlighted aberrancies in neural networks [17] and sensitization of central nervous system (CNS) processing [18]. To date, however, empirical evidence of these theories, particularly those concerning CNS processing pathways, has been somewhat limited. Quantitative sensory testing (QST) enables standardized activation of the nociceptive system and particular pathways of pain processing. Responses to these stimuli can then be quantitatively assessed through pain ratings, thresholds, and tolerances. The goal of using QST in research is to obtain a better understanding of the mechanisms involved in pain [19]. Correlations between pain catastrophizing and experimentally induced (evoked) pain would suggest that all negative stimuli, be it thoughts, emotions, or sensory pain, may be processed through a shared network. However, prior studies on this topic have yielded inconsistent results, with some studies finding significant relationships between pain intensity ratings and catastrophizing scores for certain evoked pain tasks and other studies failing to find such significant relationships [20–25].

Most prior studies of catastrophizing and pain processing have measured pain catastrophizing as a trait variable, correlating Pain Catastrophizing Scale (PCS) [9] scores with pain intensity ratings, providing us with information about pain processing in two groups of people, those with a maladaptive psychological reaction and appraisal process (catastrophizers) and those without (noncatastrophizers). The many potential factors that may differentiate these two groups of chronic pain patients confound the results and limit our ability to confidently determine the neural mechanics of pain catastrophizing and how pain catastrophizing influences pain processing.

To limit confounds present with correlational, trait-based catastrophizing studies, a few prior studies have attempted experimental catastrophizing manipulations or measured state pain catastrophizing. The PCS, as a trait measure, is intended to measure a stable pattern of catastrophizing cognitions within an individual and allow for predictions across time, while state versions of the PCS are intended to capture the impact of other variables on the individual over time, such as in prior daily diary studies of catastrophizing [26]. Healthy volunteers asked to recite items from the PCS during pain testing have shown increases in some measures of pain perception during the recitation task [27]. Another study attempted to induce pain catastrophizing in healthy participants by emphasizing the painful sensations of the cold water pressor task and the possibility of fainting from the sharp, cutting pain. This manipulation revealed modest increases in pain catastrophizing but no associated changes in pain on the cold water pressor task [28]. Authors of a third, recent study manipulated levels of pain catastrophizing through hypnotic suggestion and measured spontaneous pain intensity in chronic headache participants and evoked pain intensity in heathy controls. The authors reported that hypnotic suggestions significantly altered clinical and evoked pain intensity and pain unpleasantness in both the chronic pain and healthy participant groups and the change in pain intensity was predicted by change in pain catastrophizing scores [29].

An additional confound involves potential differences in healthy volunteers versus individuals with chronic pain, as a preexisting pain condition may uniquely impact evoked pain responses. Our study was the first to conduct a catastrophizing manipulation and measure evoked pain in a chronic low back pain sample and adopted methodology created by Darnall and colleagues of an individualized, in vivo, 10-minute pain catastrophizing induction [30]. Results from this pilot study provided preliminary evidence that negative emotional expression during the induction was associated with subsequent increases in proinflammatory cytokines (IL-6, TNF-*α*) in women with chronic pain and suggested a potential link between the induction, emotional response, and subsequent biological response. The important next step was to test how the pain catastrophizing induction might prime the body for amplified pain processing.

We sought to better elucidate the pain processing mechanisms involved in pain catastrophizing by explicitly instructing participants with chronic low back pain to catastrophize during an individualized pain catastrophizing induction and quantify subsequent changes in response to painful experimental stimuli.

**Hypothesis 1 .**

We hypothesized that state pain catastrophizing would increase following the experiment for the catastrophizing induction condition only.

**Hypothesis 2 .**

We hypothesized that, for the catastrophizing induction condition, within-subject pain intensity ratings would significantly increase and pain tolerance would significantly decrease following the experiment (QST 2) compared to preexperiment (QST 1). We hypothesized that changes in state pain catastrophizing would correlate with changes in QST pain after the experiment. These effects were expected to occur independent of baseline trait pain catastrophizing levels and baseline low back pain intensity levels. We expected that there would be no significant differences between the baseline QST results of the induction condition and the control condition.

## 2. Materials and Methods

### 2.1. Participants

The study enrolled 40 women with chronic low back pain. Twenty women were in the catastrophizing induction condition and 20 women were in the control condition (1 participant from the control condition was excluded from analysis due to missing baseline data). Women are at higher risk for developing pain and report higher levels of pain catastrophizing compared to men [31]. Women were recruited through the Stanford Systems Neuroscience and Pain Lab database and through advertisements posted on Craigslist. Participants were phone screened by the researcher for eligibility. Inclusion criteria included female sex, ≥18 years of age, an average low back pain intensity rating of ≥4 on a 0–10 scale, occurrence of low back pain ≥50% of the time, and a duration of low back pain of ≥3 months. Exclusion criteria included substance abuse problems in the past six months, suicidality, ongoing legal action regarding pain, ongoing disability claims, and pregnancy. The study visit was completed in about 4 hours; participants were compensated $20 per hour for the single study visit for a total of $80.

### 2.2. Procedures

All procedures were approved by the institutional review board at the Stanford University School of Medicine, and all patients gave informed consent prior to participation.

**Figure 1:**
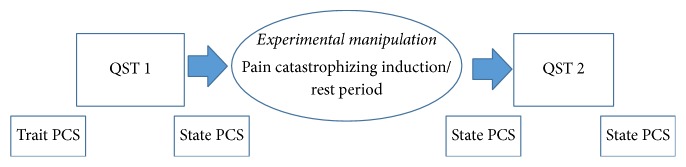
Schedule of events for study visit.

Participants began the session by completing a baseline packet of questionnaires, including demographics, pain intensity, and the Pain Catastrophizing Scale [9]. Participants then underwent a round of several QST tasks, followed by the experiment (a ten-minute pain catastrophizing induction for the induction condition or a ten-minute rest period for the control condition), and then a second round of the same QST tasks. Participants completed the state version of the Pain Catastrophizing Scale [32] at post-QST 1, postexperiment, and post-QST 2 (see Figure 1).

#### 2.2.1. Experimental Condition Allocation

Participants were not randomly assigned to condition. Participants were run in blocks, with the 20 induction condition participants first, followed by the 20 control condition participants.

#### 2.2.2. Pain Catastrophizing Induction Condition

The Pain Catastrophizing Scale [9] is composed of three subcategories, rumination, magnification, and helplessness. These three areas were directly targeted in the 10-minute pain catastrophizing induction. Study participants were directed by a female licensed clinical psychologist specializing in pain psychology (BDD) to focus on their pain (rumination), to imagine their pain worsening in the near future (magnification), and to imagine how they would be powerless in the worsening pain negatively impacting different aspects of their life (helplessness). Participants were asked to describe a “worst-case scenario” of their worsened, uncontrollable pain. The psychologist returned at the end of the study session to answer any questions the participant had about the experiment and to ensure that the participant's emotional state had returned to preinduction status. The psychologist offered resources or recommendations for coping with chronic pain on an individual basis, as needed.

#### 2.2.3. Control Condition

Participants in the control condition sat quietly for 10 minutes.

### 2.3. Measures

#### 2.3.1. Demographics

Demographics collected at baseline included age, household income, and education level.

#### 2.3.2. Pain Intensity

At baseline, participants provided ratings for current pain intensity and average pain intensity over the past 7 days on a 0–10 numerical rating scale, which has been demonstrated to be a valid method of pain intensity assessment in chronic pain research [33].

#### 2.3.3. Trait Pain Catastrophizing

The Pain Catastrophizing Scale [9] was administered at baseline only. The PCS has a three-factor structure (rumination, magnification, and helplessness) with 13 questions on a 5-point scale (0, not at all, to 4, all the time). An example item of the PCS is “I keep thinking about how much it hurts.” The PCS has been validated for use in clinical pain samples [34, 35]. In our sample, the PCS had high internal consistency (Cronbach's alpha = 0.937).

#### 2.3.4. State Pain Catastrophizing

While there is no validated tool to measure state pain catastrophizing, Edwards and colleagues developed a state version of the PCS by selecting 6 items from the PCS and altering the wording of the items for suitability in evoked paradigms [32]. The state version has the same 5-point scale as the PCS and maintains representation of the three factors in the PCS, rumination, magnification, and helplessness. In the current study, participants completed the state PCS at 3 time points: after the first round of QST, after the catastrophizing induction/10-minute rest period, and after the second round of QST. In our sample, the state version of the PCS showed high internal consistency (Cronbach's alpha = 0.914–0.957).

#### 2.3.5. QST Measures

QST tasks included weighted pin pain, pressure pain threshold, heat pain threshold and tolerance using thermal stimulation heat ramps, cold pain using a cold water pressor task, and conditioned pain modulation using the cold water pressor task and heat ramp. The same set of QST measures were administered in the same order for all participants and in the same sequence before and after the experiment (induction/10-minute rest period) in the order they are listed below. Methodology for heat, cold, pin, and pressure pain tasks was adapted from QST methods put forward by Rolke and colleagues [36] and methods for CPM were based on methods previously used by Bernaba and colleagues [37]. Additionally, 28 of the participants (13 induction condition, 15 control condition) went through a mechanical allodynia task, which was measured directly before the experiment and directly after the experiment. Two participants from the control condition were excluded from analysis, one for missing baseline data and one for failure to achieve a state of allodynia with the task. The remainder of the 40 participants lack allodynia data due to inability of the participants to tolerate the procedure (*N* = 10), session time constraints (*N* = 1), and broken thermode (*N* = 1). Methodology for the mechanical allodynia task was derived from methods previously used by Martucci and colleagues [38].

#### 2.3.6. Weighted Pin Pain

For the weighted pin task, three marks were made at midline on the participant's left arm at four, six, and eight inches from the wrist. Prior to the start of the task, participants were told that they would be asked to give a pin pain intensity rating on a scale of 0–10, 0 being no pain and 10 being worst pain imaginable, following 10 consecutive pricks (1/sec) with a 256 mN weighted pin at each mark. Participants were instructed to say “10” at any point during the task if they felt the pin pain intensity had reached 10 out of 10, even if the 10 consecutive pricks had not finished, and the task was immediately stopped. The three 0–10 pin pain intensity ratings (one for each mark) were recorded. Baseline pin pain intensity was not collected as it typically is for temporal summation tasks and this methodological limitation is expounded on in the discussion. During analysis, a pin pain density score was calculated for each location on the arm and the three pin pain density values at each administration of the task (QST 1 and QST 2) were averaged. To calculate a pin pain density estimate, baseline pin pain intensity was assumed to be 0 and the average of the pin pain intensity rating given and baseline of 0 was multiplied by the number of pricks. For example, if the participant reported a pin pain intensity rating of 4/10 after the 10th prick, pin pain density = ((0 baseline pin pain intensity + 4 pin pain intensity rating at 10 pricks)/2) *∗* 10 pricks = 20. This calculation method was used to account for some participants reporting a 10-pin pain intensity rating before the end of the 10 pricks. For example, if a participant reported 10/10 pin pain intensity after the 5th prick, pin pain density = (((0 baseline pin pain intensity + 10-pin pain intensity rating at 5 pricks)/2) *∗* 5 pricks) + (((10-pin pain intensity rating at 5 pricks + assumed 10-pin pain intensity rating at 10 pricks)/2) *∗* 5 pricks) = 75.

#### 2.3.7. Pressure Pain

Prior to the task, it was explained to the participant that the pressure would gradually increase and she should say “pain” as soon as she felt the pressure had become painful, indicating her pressure pain threshold. Pressure pain threshold was measured using a computerized circular probe pressure algometer (1 cm^2^). Increasing pressure was applied to both the right and left trapezius muscles and the right and left lower back starting from 0 pounds at a rate of approximately one pound per second while the participant was sitting up. Pressure was applied to each of the four locations three times and the second and third values for each location were averaged for analysis.

#### 2.3.8. Heat Pain

Heat was administered using a Pathway system (Medoc Advanced Medical Systems, Ramat Yishai, Israel), with a thermode secured on the left palm of the participant. The participant rated the heat pain intensity using a computerized visual analog scale (COVAS), with a left anchor of “no pain” (recorded as 0/10) and a right anchor of “worst pain imaginable” (recorded as 10/10). Heat ramps were conducted with the temperature beginning at 32.0°C and increasing at a rate of 0.3°C/second until the participant gave a 10/10 heat pain intensity rating on the COVAS or the temperature maxed out at 51.0°C. Pain threshold was considered as the temperature corresponding to a 1/10 heat pain intensity rating and pain tolerance was the temperature corresponding to the 10/10 heat pain intensity rating. Prior to the start of the task, the participant was instructed on how to use the COVAS to track the pain felt from the heat. When the participant pushed the slider all the way to the right, indicating worst pain imaginable, the test immediately stopped and the thermode instantly cooled down. The participant went through one practice trial at the beginning and then two trials where the data were recorded each time heat threshold and tolerance were assessed during the session.

#### 2.3.9. Cold Pain

The participant submerged her right foot in a tub of water cooled to 10°C without touching her foot to the bottom or sides of the tub. Prior to the start of the task, the participant was told that at several time points she would be asked for a pain intensity rating from the cold on a scale of 0–10, 0 being no pain, and 10 being the worst pain imaginable. The participant was instructed to take her foot out of the water if the cold pain reached 10 out of 10 at any point before the researcher told her the task was complete. The participant was asked for her cold pain intensity rating at 30 seconds, 60 seconds, 90 seconds, and 120 seconds. The cold pain intensity ratings were recorded and a cold pain density was calculated for the task using the same method that was described above for the weighted pin task.

#### 2.3.10. Conditioned Pain Modulation

Conditioned pain modulation (CPM) measures endogenous analgesia in humans. Activity of dorsal horn nociceptive neurons is attenuated in response to one noxious stimulus (the conditioning stimulus) and this inhibits the neural response to another noxious stimulus applied elsewhere (the test stimulus), increasing pain threshold for the test stimulus. A CPM task consists of a conditioning stimulus and a test stimulus that is measured before and during or after the application of the conditioning stimulus. CPM is considered to be detected through a change in the pain intensity rating of the testing stimulus before/after application of the conditioning stimulus [39]. CPM has been found to be commonly impaired in populations with chronic pain [40]. In this study, heat ramps were used as the test stimulus and the cold water pressor was used as the conditioning stimulus. Heat ramps were administered prior to the cold water pressor task and directly following the cold water pressor task. In analysis, change in heat pain threshold and tolerance from pre- to directly postcold water pressor were calculated.

#### 2.3.11. Mechanical Allodynia

Five minutes of heat was applied at 45° on the right volar forearm using a Medoc ATS probe. Topical capsaicin (.075%) was applied over the square stimulus area and covered with a bandage for 30 minutes. Capsaicin cream was removed and allodynia was measured along 8 orthogonal trajectories using a 256 mN von Frey filament starting from well outside the stimulus region and moving towards the stimulus site until the subject reported pain. Location of pain was marked on each trajectory. Allodynia measurements were taken immediately after the 30 minutes of capsaicin (preexperiment) and again after the induction/rest period (postexperiment). The distances from the stimulus area to point of pain detection on each of the eight axes were added together for each time point. The 256 mN von Frey filament used in the mechanical allodynia task is a different tool from 256 mN weighted pin used in the weighted pin task described above. The 256 mN von Frey filament was rated as nonpainful by study participants prior to the application of the heat and capsaicin, making it an appropriate stimulus for allodynia assessment.

### 2.4. Statistical Analysis

All analyses were conducted in RStudio with a *p* < 0.05 threshold for significance. No post hoc adjustments were made to significance values due to the pilot/exploratory nature of the study.

**Hypothesis 1 .**

Linear mixed models were performed to examine the effect of condition (induction and control) on state PCS scores over time and to test the significance of changes in state PCS scores across conditions.

**Hypothesis 2 .**

Linear mixed models were performed to examine the effect of condition (induction and control) on QST pain intensity ratings over time to test the hypothesis that, for the induction condition, within-subject pain intensity ratings would significantly increase and pain tolerance would significantly decrease following the pain catastrophizing induction (QST 2) compared to preinduction (QST 1). State PCS scores were added as predictors in any model where the change in the QST variable following the induction was significant. This step was added to determine whether inclusion of changes in state PCS would statistically account for induction-related changes in QST pain.

## 3. Results

### 3.1. Demographic Characteristics

Participants had a mean age of 51 (SD = 12.0), and an independent samples *t*-test found no significant difference in age between the pain catastrophizing induction condition and control condition (*t* = 2.06, *p* = 0.051). The median education level in the sample was a two-year college degree/vocational certificate, and a chi-squared test revealed no significant difference in education level between the induction condition and control condition (*χ*^2^ = 8.5, *p* = 0.08). A chi-squared test found a significant difference in household income between the two conditions (*χ*^2^ = 15.3, *p* = 0.03), with the induction condition having a median household income of $20,000–$29,000 and the control condition having a median household income of $80,000–$89,000. Participants reported an average back pain intensity over the past 7 days of 6.0 (SD = 2.1), and an independent samples *t*-test revealed no significant difference between the induction condition and control condition (*t* = 1.86, *p* = 0.07). Participants had an average current back pain intensity of 4.8 (SD = 2.4) and independent samples *t*-test revealed a significant difference between the induction condition (mean = 5.6, SD = 2.1) and the control condition (mean = 4.0, SD = 2.6). Due to condition differences in baseline current back pain intensity, this variable was included as a covariate in subsequent analyses.

### 3.2. Experimental Manipulation Check

Averages were calculated for each participant for baseline trait PCS, post-QST 1/preexperiment state PCS, postexperiment state PCS, and post-QST 2 state PCS. A total of 9 individual items for the state or trait PCS were not completed across the participant sample (<1% missing data rate); in order to avoid calculating artificially lower PCS scores, averages were computed instead of sum scores. The trait PCS has a total of 13 items, each on a 0–4 scale, resulting in a maximum average score of 4. State PCS has a total of 6 items, also each on a 0–4 scale, resulting in a maximum average of 4. An independent samples *t*-test revealed a significant difference in baseline trait PCS scores between the induction condition and control condition (*t* = 2.04, *p* = 0.049). Due to condition differences in trait PCS at visit baseline, this variable was included as a covariate in subsequent analyses. Independent samples *t*-tests revealed no condition difference in preexperiment state PCS scores between the induction condition and control condition and did reveal a significant condition difference in state PCS scores following the experiment (*t* = 4.66, *p* < 0.001) and following the second round of QST (*t* = 2.13, *p* = 0.04), signaling the effectiveness of the induction at increasing state catastrophizing levels (see Table 1).

### 3.3. Association between Experimental Condition and State PCS

**Figure 2:**
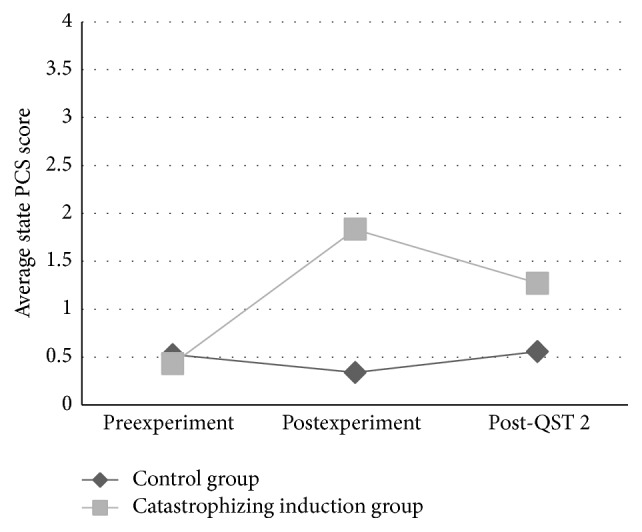
State PCS score over time by experimental condition. There was a significant interaction of time and condition on state PCS controlling for baseline trait PCS and baseline current back pain intensity (unstandardized *β* = −0.39, *p* = 0.02), with significantly larger increases in PCS scores after the experiment in the pain catastrophizing induction condition compared to the control condition.

There was a significant interaction of time and condition on state PCS controlling for baseline trait PCS and baseline current back pain intensity (unstandardized *β* = −0.39, *p* = 0.02), with significantly larger increases in PCS scores after the experiment in the induction condition compared to the control condition (see Figure 2).

### 3.4. Association between Experimental Condition and QST

**Figure 3:**
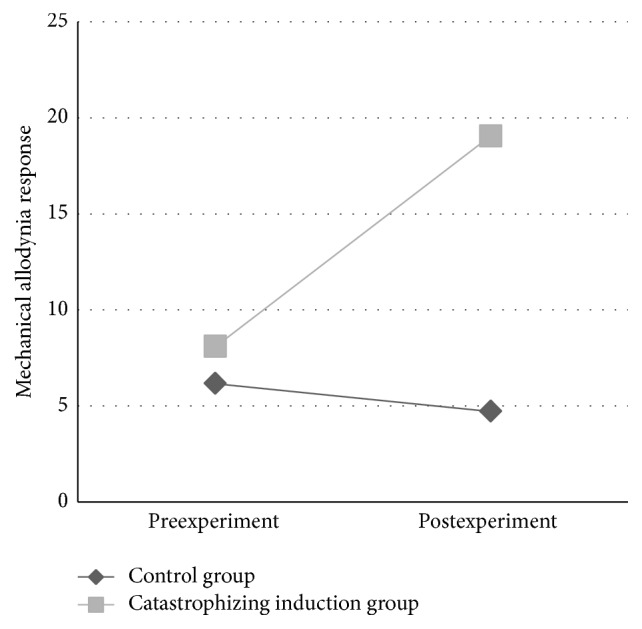
Mechanical allodynia response over time by experimental condition. The interaction of time and condition on mechanical allodynia, controlling for baseline trait PCS and baseline current back pain intensity, was significant (unstandardized *β* = −6.20, *p* < 0.001), with allodynia being significantly greater in the induction condition following the experiment.

**Figure 4:**
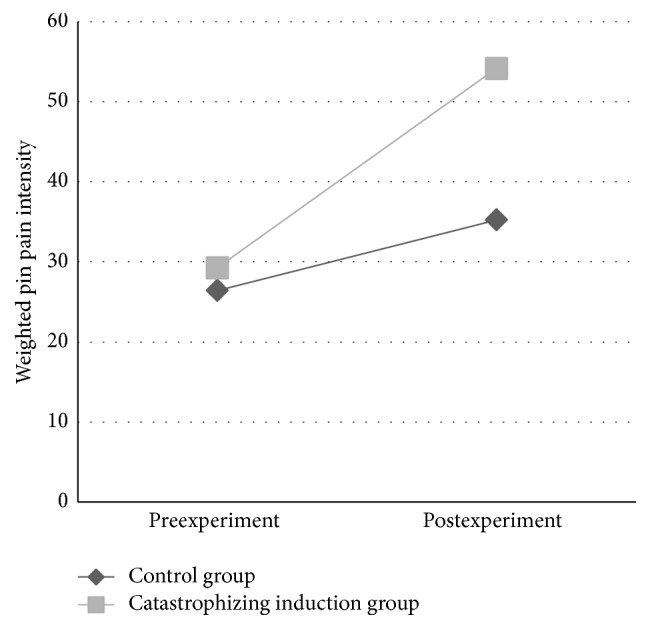
Weighted pin pain intensity over time by experimental condition. The interaction of time and condition on weighted pin pain intensity, controlling for baseline trait PCS and baseline current back pain intensity, was significant; weighted pin pain was significantly higher in the pain catastrophizing induction condition after the experiment compared to before the experiment (unstandardized *β* = −7.85, *p* = 0.01).

A table of means and standard deviations for all QST assessments for the induction and control condition across time can be found in Table 2. In general, there were no significant differences between baseline QST results of the induction and control condition with the exception of the induction condition having lower baseline CPM heat threshold (unstandardized *β* = 2.22, *p* = 0.01) and the induction condition having higher cold pain (unstandardized *β* = −203.26, *p* = 0.05). As predicted, the interaction of time and condition on mechanical allodynia, controlling for baseline trait PCS and baseline current back pain intensity, was significant (unstandardized *β* = −6.20, *p* < 0.001), with allodynia being significantly greater in the induction condition following the experiment (see Figure 3). The interaction of time and condition on weighted pin pain, controlling for baseline trait PCS and baseline current back pain intensity, was also significant; weighted pin pain was significantly higher in the induction condition after the experiment compared to before the experiment (unstandardized *β* = −7.85, *p* = 0.01) (see Figure 4). The interaction of time and condition on QST pain was not significant for any of the other QST tasks administered, including heat pain threshold, heat pain tolerance, pressure pain threshold, CPM, and cold pain tolerance. Inclusion of state PCS scores did not significantly change the induction-related changes in allodynia; the condition-by-time interaction remained significant (unstandardized *β* = −5.32, *p* = 0.002) and state PCS change was not a statistically significant predictor in the model (unstandardized *β* = 2.30, *p* = 0.08). When scores for state PCS were included as a predictor for change in pin pain, the condition-by-time interaction was no longer significant (unstandardized *β* = −6.01, *p* = 0.08), but the effect of state PCS was also nonsignificant in the model (unstandardized *β* = 4.69, *p* = 0.12). For exploratory purposes, we ran the models with state PCS and baseline current pain as predictors but omitted baseline trait PCS as a predictor due to suspected collinearity between baseline PCS scores and state PCS scores. For allodynia, this model yielded a significant time by condition interaction (unstandardized *β* = −5.28, *p* = 0.002) and state PCS was a marginally significant predictor (unstandardized *β* = 2.40, *p* = 0.05). For pin pain intensity, this model yielded a nonsignificant time by condition interaction (unstandardized *β* = −5.36, *p* = 0.11) and state PCS was significant (unstandardized *β* = 6.66, *p* = 0.01), suggesting that state PCS scores accounted for a significant degree of the effect of the experimental condition on pin pain intensity.

## 4. Discussion

The current study was intended to gauge the effects of a personalized catastrophizing induction on QST in a chronic pain sample, extending previous investigations of catastrophizing and response to evoked pain paradigms. Although the catastrophizing induction appeared to increase state pain catastrophizing levels, the effects of the experiment on QST pain were not particularly robust and limited to certain types of evoked pain. Experiment-related differences in QST were only detected for two of the tasks administered, weighted pin pain and mechanical allodynia. Mechanical allodynia is considered to be a main feature of central sensitization and is defined by heightened pain to mechanical nociceptive stimuli [41]. Temporal summation refers to the increase in perceived pain from a repetitive, noxious stimulus [42]. Temporal summation represents wind-up, a physiological phenomenon of central sensitization where increased firing of spinal neurons amplifies pain processing and can be facilitated through a variety of noxious stimuli, such as heat, pressure, and pinprick [43]. To the extent that the findings of this pilot study may reflect a relationship between state levels of catastrophizing and experimentally induced pain, the strongest potential relationship appears to be between state pain catastrophizing and forms of mechanical pain that may reflect central sensitization.

Similar to the results of our primary analyses, the results for our secondary analyses were equivocal, in that state pain catastrophizing levels only appeared to predict pin pain and not allodynia and only when baseline trait PCS was not included as a predictor in the model. We had no a priori reason to expect only the pin and allodynia tasks would relate to induced catastrophizing states. Prior literature has mixed results on which types of QST tasks correlate with pain catastrophizing levels, however some prior findings do appear to align with the results found in this pilot. Previous studies have found a significant relationship between PCS and temporal summation pain in healthy subjects [32] and in participants with chronic low back pain [21] and a more recent study found that catastrophizing mediated the relationship between dispositional optimism and temporal summation in a sample of participants with knee osteoarthritis [23]. We are unable to make a direct comparison between the results of these prior studies and our study because the current study did not collect a baseline pain for the weighted pin task which is required to most appropriately calculate temporal summation of pain. However, with this in mind, we may hypothesize that the weighted pin task in this study was activating wind-up of pain.

Salomons and colleagues found that a brief cognitive-behavioral intervention reduced secondary hyperalgesia compared to a control condition and change in secondary hyperalgesia in the intervention condition was significantly correlated with change in Pain Catastrophizing Scale score [44]. In our study, we conducted the opposite manipulation, testing whether a negative induction would correlate with increases in mechanical allodynia and our results supported this hypothesis. The Salomons study instilled an enhanced degree of healthy cognitive reappraisal of pain and found reduced in their QST task assessing central sensitization, and our study induced catastrophic thoughts about pain and found a heightened degree of allodynia, similarly assessing central sensitization. The recent study by Kjøgx and colleagues was able to successfully manipulate pain catastrophizing in both positive and negative directions through hypnotic suggestion and found these manipulation significantly influenced pain levels [29]. Our study results support the findings of these previous studies demonstrating a relationship between pain intensity ratings and immediate cognitive appraisal of pain.

There are several limitations that should be noted. A significant limitation of the study is the block assignment to condition procedure that was employed. The lack of random assignment to experimental condition may have resulted in baseline group differences that serve as potential confounds and complicates interpretation of the results. While a strength of this study involved uniformity of administration for the pain catastrophizing induction and QST for all participants, the administrator of the QST tasks was not blinded to the condition or the hypotheses of the study. Additionally, some potential confounds, such as medication usage, were not assessed or controlled for in analysis. While the weighted pin task was likely capturing wind-up signal, we cannot conclusively say is a measure of temporal summation due to the lack of collection of a baseline pain intensity rating after the first pinprick.

As with all pilot studies, the small sample size of the current study stands as a limitation. Given that the study was intended as a signal-finding effort, we did not control for multiple comparisons given the small sample size. As a result, there is an increased risk of Type 1 error that should be acknowledged. As this was a preliminary investigation with a limited number of participants, we urge replication of these findings in a larger sample.

Although there remains a need to replicate our findings using a random assignment model with a larger sample size, we have found some degree of evidence that there may be a relationship between catastrophizing and central sensitization of pain. Additional effects may exist within a “high catastrophizer” subgroup, not captured here due to sample size and relatively low levels of catastrophizing. Future research could also investigate the impact of the catastrophizing induction on other QST measures not used in this study. It would be of particular interest to see if other tasks involving central modulation yield significance while other tasks involving other pain mechanisms do not, as our findings signal.

In future investigations, an inclusion of a final round of QST taking place a few hours after the experiment may reveal greater effects. Pilot data produced by Darnall et al. (2010) revealed that circulating proinflammatory cytokines were increasing 2.5 hours after the pain catastrophizing induction, thereby suggesting a formidable delay in biological response. As such, it is possible that our final QST measurement was conducted at an insufficient latency to produce biological changes that may subsequently modulate pain perception.

Finally, we recommend that future studies include male participants for a sex comparison given the documented sex differences in both catastrophizing tendencies and pain processing [31].

## 5. Conclusions

The prior literature examining the impact of catastrophizing on evoked pain are somewhat inconsistent and the relationship has rarely been examined in clinical populations. The results from our pilot study suggest that pain catastrophizing may be associated with heightened pain on some QST tasks but not others. The signals this study found for the mechanical allodynia task and the weighted pin task point towards a circulating theory that catastrophizing may contribute to chronic pain by functioning through central processing of pain by way of increased facilitation of nociceptive signals. Prior imaging work has shown that catastrophizing is linked to increased activation in cerebral regions responsible for anticipation, attentional, and emotional aspects of pain, including the medial frontal cortex, dorsolateral prefrontal cortex, anterior cingulate cortex, and insula, facilitating heightened pain processing [17, 45, 46]. Evidence suggests that the increased signaling in the brain in high catastrophizers may increase activity at the spinal cord level, leading to central sensitization and pain wind-up [46, 47]. Enhanced excitability in the central nervous system is regularly observed in people with chronic pain [41] and the investigation into what factors are influencing this dysregulation of pain modulation is important for understanding the chronification of pain and how to best treat it. This work points to the utilization of psychological modalities to prevent and treat central sensitization in chronic pain patients.

## Figures and Tables

**Table 1:** Trait and state PCS scores.

	Pain catastrophizing induction condition	Control condition
Baseline trait PCS mean ^*∗*^(*p* = 0.049)	1.72 (1.0)	1.12 (0.9)
Post-QST 1 and preexperiment state PCS mean (*p* = 0.7)	0.43 (0.7)	0.53 (0.7)
Postexperiment state PCS mean ^*∗*^(*p* < 0.001)	1.83 (1.3)	0.34 (0.6)
Post-QST 2 state PCS mean ^*∗*^(*p* = 0.04)	1.27 (1.3)	0.56 (0.8)

^*∗*^Statistical significance (*p* < 0.05).

**Table 2:** Means and standard deviations for all QST assessments for the induction and control conditions at QST 1 and QST 2.

	QST 1		QST 2	
	Induction condition	Control condition	Induction condition	Control condition
Pin pain density	29.25 (19.64)	26.22 (17.08)	54.17 (31.12)	35.44 (21.23)
Trapezius pressure pain	5.51 lbs (2.02 lbs)	7.40 lbs (4.56 lbs)	5.15 lbs (2.05 lbs)	7.13 lbs (4.31 lbs)
Low back pressure pain	7.17 lbs (3.08 lbs)	9.16 lbs (4.31 lbs)	6.51 lbs (2.93 lbs)	9.05 lbs (4.39 lbs)
Heat pain threshold	41.60°C (3.48°C)	42.25°C (4.12°C)	39.37°C (3.60°C)	40.76°C (3.82°C)
Heat pain tolerance	46.25°C (2.50°C)	46.53°C (1.64°C)	44.43°C (2.86°C)	45.21°C (2.48°C)
Cold pain density	820.88 (278.86)	655.26 (297.06)	927.30 (255.48)	738.03 (264.26)
CPM threshold	−0.51°C (2.03°C)	1.50°C (2.73°C)	1.74°C (3.39°C)	2.30°C (2.92°C)
CPM tolerance	2.54°C (13.7°C)	0.73°C (1.35°C)	0.89°C (2.09°C)	1.93°C (1.36°C)
Mechanical allodynia	8.1 cm (10.06 cm)	6.16 cm (6.33 cm)	19.05 cm (15.14 cm)	4.72 cm (6.31 cm)

*Note*. For a better understanding of the values, refer to the “QST Measures” section in Methods. ^*∗*^Statistical significance (*p* < 0.05).
